# Obituary

**Published:** 1910-02-05

**Authors:** 


### Obituary.

We have to record the death of Mr. William Ilbert Hancock, at the age of thirty-six. He studied at Guy's, and took his diploma in 1896, becoming a Fellow of the R.C.S. two years later. He held several appointments as surgeon or assistant surgeon at different ophthalmic hospitals, among others at the Central London Ophthalmic Hospital, where he was senior assistant surgeon and clinical assistant. He also contributed several papers to the London Ophthalmic Hospital Reports.

